# Terpenoids from Weedy Ricefield Flatsedge (*Cyperus iria* L.) Are Developmentally Regulated and Stress-Induced, and have Antifungal Properties

**DOI:** 10.3390/molecules23123149

**Published:** 2018-11-30

**Authors:** Yifan Jiang, Bonnie H. Ownley, Feng Chen

**Affiliations:** 1 College of Horticulture, Nanjing Agricultural University, Nanjing 210095, China; jiangyifan@njau.edu.cn; 2 Department of Plant Sciences, University of Tennessee, Knoxville, TN 37996, USA; 3 Department of Entomology and Plant Pathology, University of Tennessee, Knoxville, TN 37996, USA; bownley@utk.edu

**Keywords:** antifungal, biotic stress, flatsedge, methyl jasmonate, ricefield, sesquiterpenoid, weed

## Abstract

Ricefield flatsedge (*Cyperus* *iria* L.), a troublesome weed in rice production, actively adapts to ecological niches. In this study, terpenoids were identified as the dominant compounds from organic extracts of *C. iria* leaves. To understand the role of terpenoid production in plant development and resistance to abiotic and biotic stresses, the dynamics of terpenoid production at different developmental stages, and the regulation of these compounds by stresses were determined. Terpenoid production exhibited temporal and spatial specificity. During vegetative growth, the total concentration of sesquiterpenoids increased and reached a maximum at 70 d after germination, and then decreased until the emergence of inflorescence. Monoterpenoids were only detected from leaves 90 d after germination. During reproductive growth, the total concentration of sesquiterpenoids increased dramatically and mainly accumulated in inflorescences, indicating that the sesquiterpenoids were primarily produced in newly formed and actively growing tissues. The total amount of monoterpenoids, mostly accumulated in flowers, increased until 130 d after germination. Furthermore, accumulation of sesquiterpenoids in leaves was promoted significantly by methyl jasmonate (MeJA) and drought treatment. Infestation by beet armyworm (*Spodoptera exigua*, BAW) promoted the emission of total sesquiterpenoids significantly and induced the production of more monoterpenoids and sesquiterpenoids specifically. Furthermore, volatiles from *C. iria* leaves had an anti-fungal effect on *Fusarium graminearum*. The implications of our findings on the biosynthetic pathways leading to the production of sesquiterpenoids in *C. iria* as well as their potential as fungicides are discussed.

## 1. Introduction

Weeds are undesirable plant species that reproduce quickly, disperse widely, establish a population outside their native habitat readily, and resist eradication once established. These properties offer them an advantage over more desirable species like crop, lawn or landscape plants by competing with them for limited resources. Weeds can become dominant and vigorous in new environments, which is mainly attributed to the strategies they employ to actively adapt to new ecological niches [1]. Chemical defense is one of the ways that weeds avoid infestations of herbivores and interference from indigenous plants. For example, weed roots may produce allelopathic chemicals that are lethal to more desirable indigenous or crop plants, while the aerial part produces specific secondary metabolites, especially volatiles, as a tool for defense against biotic and abiotic stresses [2].

Plant secondary metabolites constitute a broad spectrum of low molecular weight compounds involved in myriad complex processes in plants. Some secondary metabolites serve as attractants for pollinators and seed dispersing animals; some protect plants from attack by insects and pathogens; and others are involved in plant survival during abiotic stress [3,4,5]. Among the diverse secondary metabolites, terpenoids are the largest class produced in the plant kingdom and play an important role in the defense against other organisms [6,7]. It has been reported extensively that terpenoids play a role in attracting natural enemies of feeding herbivores for indirect defense [8] and display antimicrobial and antifungal activity [9,10,11].

*Cyperus* is a large genus of about 600 species of sedges, distributed throughout all continents in both tropical and temperate regions. Some tuber-bearing species, like purple nutsedge (*Cyperus rotundus* L.), yellow nutsedge (*Cyperus esculentus* L.) and ricefield flatsedge (*C*. *iria*), are considered invasive weeds leading to tremendous yield loss of rice due to their aggressive characteristics [12]. Complex terpenoids have been characterized from essential oils of several species of *Cyperus*, and related genera, including *C. rotundus*, *C. esculentus*, *Kyllinga brevifolia* Rottb. (formerly *C. brevifolius*) and *Kyllinga nemoralis* (Hutch & Dalz.) (formerly *C. kyllingia*) [13,14,15], suggesting that terpenoids may play an important role in defense against stress of members of *Cyperaceae*. Extracts and compounds from *C. iria*, a representative sedge species, have been used in traditional Chinese medicine for therapy of rheumatism, injury and fracture in China for a long time [16]. Furthermore, it has been documented that *C. iria* contains some active allelochemicals [17]. Despite this information, little is known on developmental regulation of terpenoids and how terpenoid production is affected by stresses in weedy *Cyperus*.

In this study, we chose *C. iria* as a model system to gain insight into the terpenoid-based chemical defense of weeds. Our first goal was to determine what terpenoids *C. iria* plants produce utilizing organic extraction coupled with gas chromatography-mass spectrometry (GC-MS) analysis. Secondly, once terpenoids were detected, we were interested in determining how they are regulated at different plant developmental stages, and when grown under different stresses, including drought, physical wounding, and insect infestation. The third objective of this study was to determine whether the terpenoids have any direct biological function, especially an anti-fungal effect. This is the first report on production of terpenoids at different developmental stages, as well as their regulation in response to stresses in *C. iria*. This information will likely be useful in developing new strategies for controlling *C. iria*.

## 2. Results and Discussion

### 2.1. Temporal and Spatial Differences in Terpenoid Content

**Figure 1:**
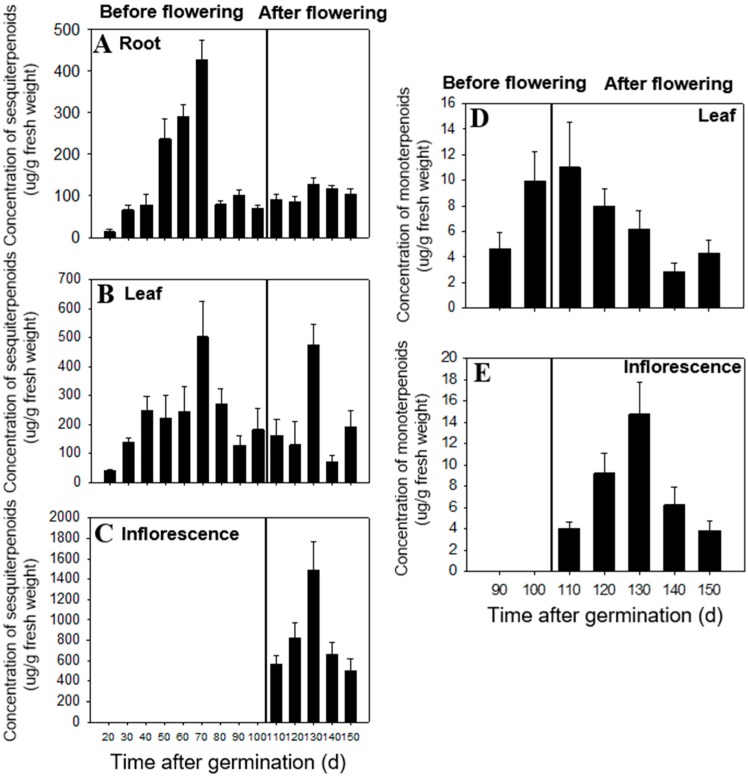
Concentration of total sesquiterpenoid (**A**–**C**) and monoterpenoid (**D**,**E**) compounds in roots, leaves, and flowers of *C. iria* at different developmental stages. Standard error is denoted for each bar.

In this study, organic extraction followed by GC-MS analysis showed that all tissues (leaf, root, and inflorescence) of *C. iria* are rich in terpenoids, which is consistent with reports on the chemical composition of essential oils in other species of *Cyperus* [12,13,14]. Terpenoid content of extracts in different tissues at different developmental stages was analyzed. We found that production and biosynthesis of terpenoids in *C. iria* displayed temporal and spatial specificity. During the immature stage (within 110 days after germination), the total concentration of sesquiterpenoids increased to a maximum level at 70 days after germination and then decreased until inflorescence occurred. The sesquiterpenoids mainly accumulated after 40 days, in roots during 50 days to 70 days, and in leaves during 80 days to 100 days after germination (Figure 1A,B). During the mature stage (110 days after germination), the total concentration of sesquiterpenoids increased dramatically and mainly accumulated in inflorescences (Figure 1C). The majority of sesquiterpenoids from *C. iria*, including β-elemene, *E-*β-caryophyllene, α-humulene, α-bergamotene, α-farnesene, elemol, hedycaryol, β-eudesmol, α-eudesmol, and γ-eudesmol, could be identified from all developmental stages in a constitutively accumulated pattern, while production of others showed temporal and spatial specificity. Germacrene D and α-copaene were only identified from inflorescences after 110 days; α-gurjunene and germacrene B were only present in roots after 70 days; and farnesol was only identified after 30 days from roots (data not shown).

Monoterpene compounds were not identified prior to 90 days after germination from any tissues, but at 90 days, β-pinene, limonene and β-ocimene started to accumulate in leaves (Figure 1D). In the mature stage of plant development, the total amount of monoterpenes in inflorescences increased until 130 days, and then decreased (Figure 1E). The percentage of monoterpenes in different tissues demonstrated that the monoterpenes are mainly produced in inflorescences after maturation. Production of individual monoterpenes also exhibited temporal and spatial specificity. For example, cis-β-ocimene was found in inflorescences and leaves after plant maturation, while limonene was only found in leaves after maturation. In addition, β-pinene was detected only in leaves after 90 days and in inflorescences at 120 days after germination. No monoterpenes were identified from roots during the 150-day plant production period (data not shown).

Temporal and spatial specificity in the content of JH III, a structurally-related sesquiterpenoid, has also been reported in *C. iria* [18,19]. Eighty-five percent of the total insect juvenile hormone (JH) detected in *C. iria* was found in the roots during the immature stage of plant development, which is consistent with the temporal and spatial distribution of sesquiterpenoids discovered in this study (Figure 1A) [20]. We postulate that the majority of the sesquiterpenoids in *C. iria* are produced in all the tissues throughout the lifetime of the plant, except for some individual sesquiterpenoids (i.e., germacrene D, α-copaene, α-gurjunene, germacrene B, and farnesol), which are only synthesized in juvenile and meristematic tissues. Monoterpene production, however, differed from production of sesquiterpenoids because it accumulated only in the above-ground tissues (in leaves during the immature stage and in flowers during the mature stage). This suggests that sesquiterpenoids in the roots may be correlated to the biosynthesis of JH III, and may play a special role in the defense of underground roots. Because self-pollination is a frequent event in *C. iria*, the main accumulation of sesquiterpenoids and monoterpenes in the reproductive tissues during the mature stage suggests an alternative biological role of terpenoids in defense, rather than in pollinator attraction.

### 2.2. Regulation of Terpenoid Accumulation by Abiotic Stress

**Figure 2:**
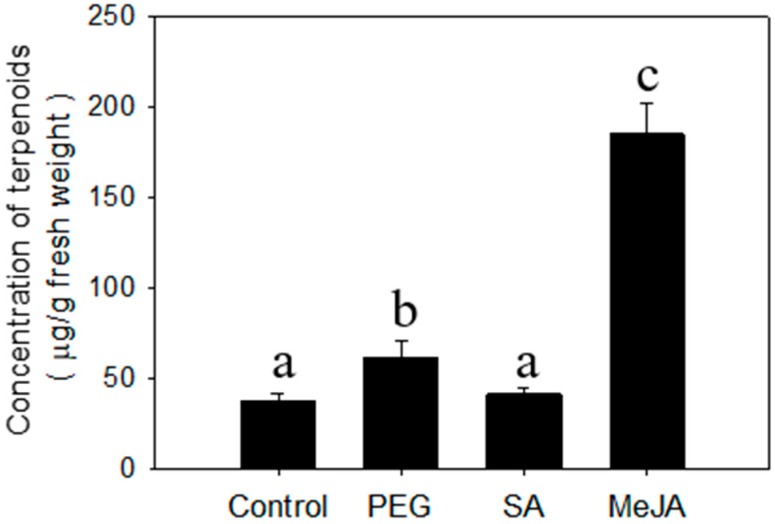
Concentration of total terpenoids from *C. iria* leaves after treatment with salicylic acid (SA), methyl jasmonate (MeJA), and Polyethylene Glycol 8000 (PEG) treatment. Standard error is denoted for each bar. Different letters (a, b and c) denote significant differences between treatments at *P* ≤ 0.05 according to an F-protected Tukey’s test.

*Cyperus iria* plants at 20 days after germination were used to conduct the chemical treatment assays. Salicylic acid (SA) had no significant effect on accumulation of total terpenoids in leaves (Figure 2). In contrast, the concentration of terpenoids was promoted significantly by MeJA in leaves (Figure 2). Moreover, one monoterpene (linalool) and one sesquiterpenoid (nerolidol) were specifically induced by treatment with MeJA (Table 1). While there were no terpenoids elicited by PEG that were not present in the control, the concentrations of several sesquiterpenes (*E-*β-caryophyllene, α-humulene, α-bergamotene, α-farnesene, elemol, hedycaryol, and γ-eudesmol) were significantly greater than the control, which accounts for the increase in total terpenoids. (Figure 2; Table 1). 

### 2.3. Regulation of Terpenoid Accumulation by Biotic Stress

**Figure 3:**
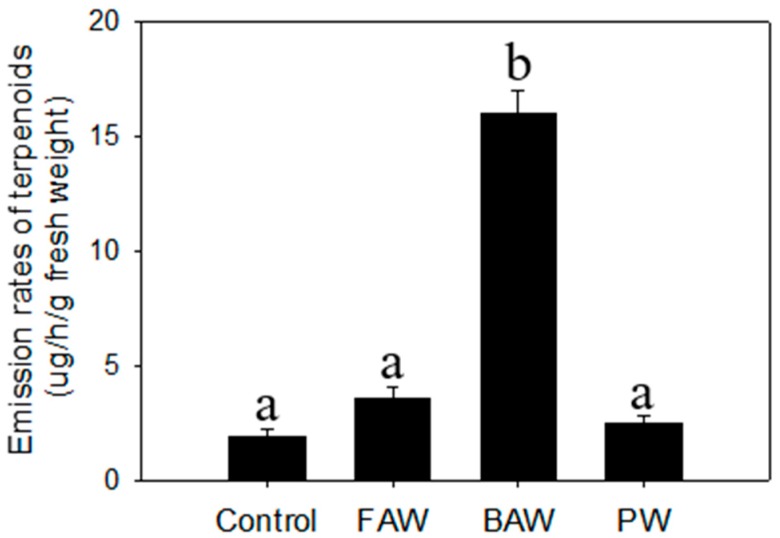
Emission rates of total terpenoids from physical wounding (PW), fall armyworm (FAW) and beet armyworm (BAW) from *C. iria* leaves. Standard error is denoted for each bar. Different letters (a and b) denote significant differences between treatments at *P* ≤ 0.05 according to an F-protected Tukey’s test.

**Figure 4:**
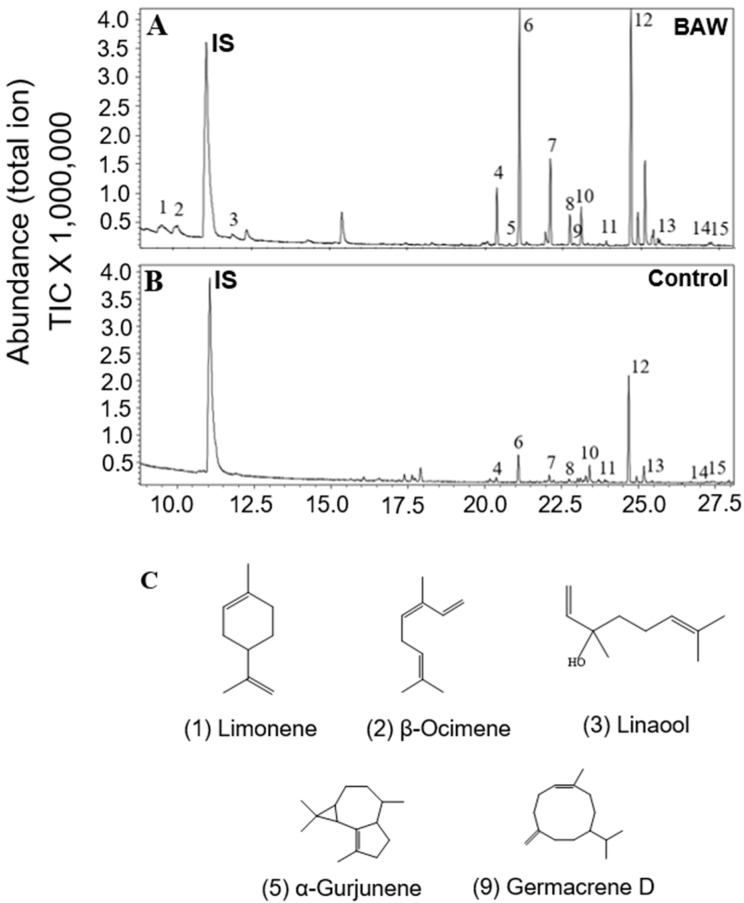
Profile of terpenoids from leaves of BAW infested plants (**A**) and control plants (**B**) by headspace volatile collection and GC-MS analysis. 1, Limonene; 2, β-Ocimene; 3, Linalool; 4, β-Elemene; 6, *E-*β-Caryophyllene; 7, α-Humulene; 8, α-Bergamotene; 9, Germacrene D; 10, α-Farnesene; 11, Elemol; 12, Hedycaryol; 13, γ-Eudesmol; 14, β-Eudesmol; and 15, α-Eudesmol. Unlabeled peaks are not terpenoids. The internal standard is represented by IS. The chemical structure of terpenoid compounds specifically induced by BAW infestation (**C**).

Unlike the organic extraction from plants exposed to abiotic stresses, we employed headspace volatile collection to plants treated with biotic stresses to investigate aerial volatiles that might play important biological roles, especially involving indirect defense. The results indicated that there were no evident differences detected in the profile and total emission rates of terpenoids from *C. iria* leaves after infestation of fall armyworm (FAW) and physical wounding. For individual terpenoid compounds, there was a significant increase in the emission rates of *E-*β-caryophyllene, α-bergamotene, α-farnesene, and α-eudesmol after physical wounding, and of *E-*β-caryophyllene, γ-eudesmol, and β-eudesmol following infestation of FAW. Interestingly, the emission rates of total terpenoids in leaves was promoted significantly by infestation of beet armyworm (Figure 3). Some monoterpenes (limonene, cis-β-ocimene, linalool) and sesquiterpenoids (germacrene D, α-gurjunene), which have been reported to be induced by other species of herbivores to attract natural enemies of the insect pest for indirect defense, were emitted specifically following infestation of BAW (Figure 4; Table 1).

As a vigorous weed in rice fields, *C. iria* plants are inevitably exposed to various stresses, like drought, insect infestation, and pathogens that also cause stress to rice. However, few symptoms caused by either biotic or abiotic stresses could be discerned in wild *C. iria* plants, compared to rice, suggesting that *C. iria* has typical characteristics of weeds that exhibit strong resistance to various stresses. Unlike terpenoid emission in other monocot species, including rice, sorghum and maize, the majority of terpenoids in *C. iria* are constitutively produced without stress. Thus, whether the terpenoids could be qualitatively and quantitatively elicited by abiotic and biotic stresses in *C. iria* was unknown. We found that an infestation of BAW increased emission rates of terpenoids and induced production of several terpenoids (limonene, cis-*β*-ocimene, linalool, germacrene D and α-gurjunene) in the leaves of *C. iria*. These compounds have been reported to be induced by herbivores in other monocot crops, and to attract natural enemies for indirect defense against insects [21,22,23,24]. Therefore, we speculate that terpenoids induced by BAW infestation may be a strategy employed by *C. iria* to attract natural enemies for indirect defense against insect pests.

Moreover, the biosynthesis of terpenoids in plant species is typically regulated by several factors, such as exogenous hormones related to signal transduction. Based on our results, application of exogenous MeJA can also promote accumulation of sesquiterpenoids. The profile of terpenoids by exogenous MeJA application is basically consistent with BAW infestation (Table 1). Therefore, we speculate that biosynthesis of sesquiterpenoids probably depends on the JA pathway, which is consistent with previous reports in other plant species [25]. Collectively, this evidence indicates that *C. iria* leaves have a large capacity to respond to jasmonic acid signaling pathway elicitors, suggesting that biotic stress can modify terpenoid biosynthesis in *C. iria*.

### 2.4. Cluster Analysis of Sesquiterpenoids after Different Treatments

**Figure 5:**
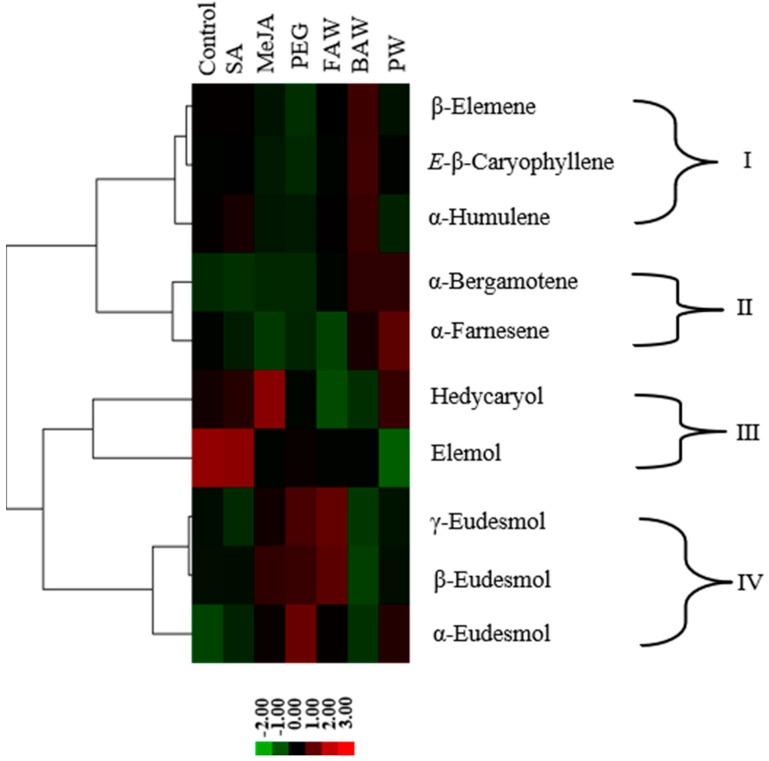
Cluster analysis of sesquiterpenoids. Hierarchical cluster analysis was performed based on the concentration of chemicals from leaves of *C. iria* under seven conditions, including control, SA, MeJA, PEG, PW, FAW and BAW, respectively. Sesquiterpenoids were grouped into four clades (clade I–IV).

Sesquiterpenoids were identified as the main and most diverse component in the extract of *C. iria*. To understand the molecular basis for the overall abundance and diversity of sesquiterpenoids, cluster analysis was performed using concentrations or emission rates of sesquiterpenoids across different treatments to predict the genetic complexity of sesquiterpenoids biosynthesis in *C. iria*. Four clades were obtained that contained three, two, two, and three sesquiterpenoids, respectively (Figure 5). β-elemene, *E-*β-caryophyllene, and α-humulene were grouped in clade I, while α-bergamotene and α-farnesene were grouped as clade II. Elemol and hedycaryol were grouped together as clade III, while γ-eudesmol, β-eudesmol, and α-eudesmol were grouped in clade IV.

Important qualitative and quantitative variations in the content of terpenoids were observed by induction of different types of stress, especially by MeJA treatment and BAW infestation, which reflects changes in the regulation of expression of terpene synthase (TPS) genes. Hence, differences in product profiles of different synthases, and differences in expression regulation could be used to identify the minimum number of synthases responsible for the overall terpenoid production spectrum. Whereas some sesquiterpenoid synthases form a single product, most of them catalyze the formation of multiple products that appear consistent in relative proportions. Because the compounds in each clade showed similar patterns of accumulation, they are likely the products of a single enzyme [26,27]. Based on a hierarchical cluster analysis, we separated four clades (Figure 5), each characterizing a similar pattern of sesquiterpenoid accumulation following abiotic or biotic stress, suggesting that the sesquiterpenoids in each clade were synthesized by at least one different TPS enzyme. In our study, each of the clades were characterized by a specific type of cyclization, supporting the assumption that the terpenoid products of each clade were formed by one TPS. Based on this reasoning, at least four TPS enzymes are involved in the production of sesquiterpenoids in *C. iria* under control and stress-elicited conditions. Until now, no TPS genes expressed in *C. iria* leaves have also been functionally characterized. The application of cluster analysis as a tool will facilitate identification of putative TPS genes responsible for the terpenoids produced by *C. iria*.

### 2.5. Anti-Fungal Effect of Volatiles Emitted from Leaves

**Figure 6:**
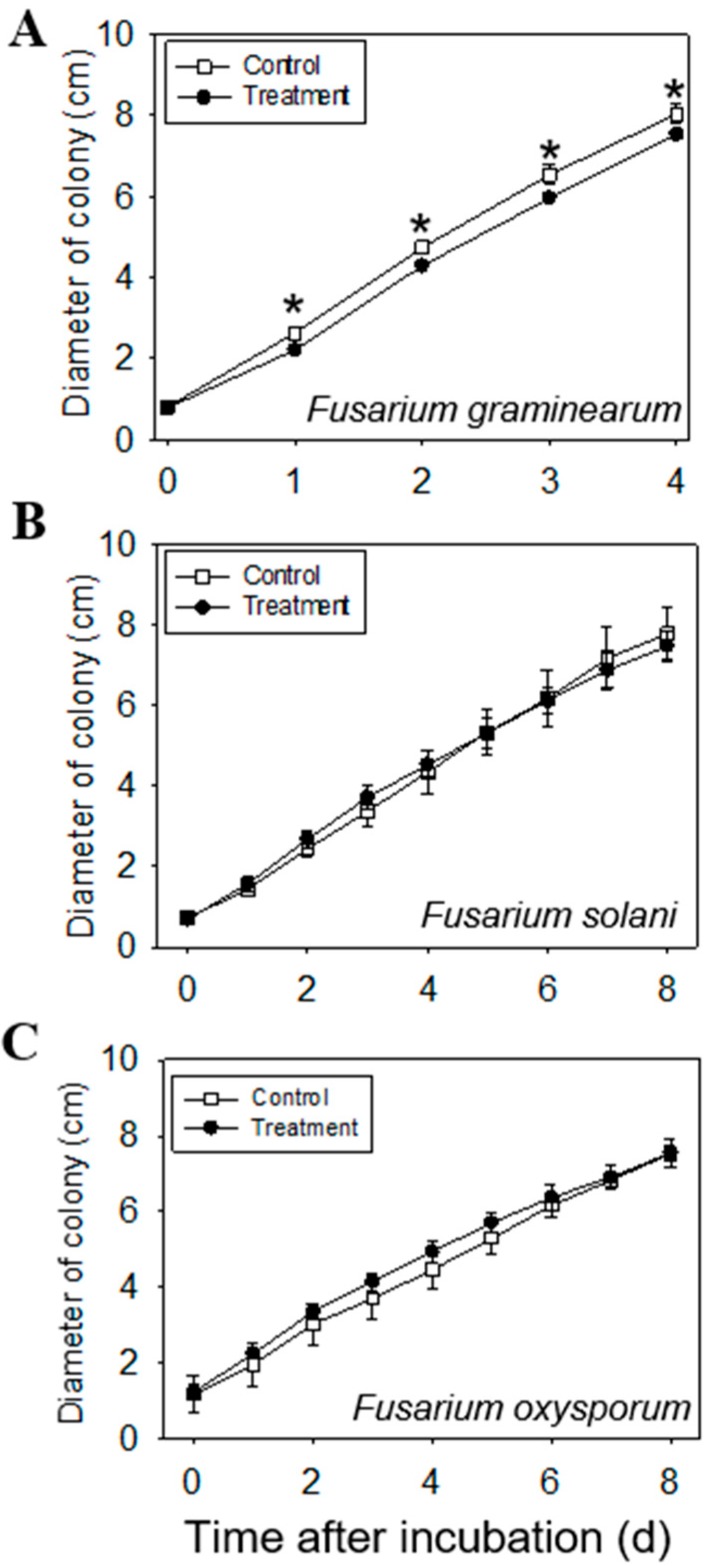
Daily antifungal effect of volatiles from *C. iria* leaves to *Fusarium* species until the mycelium reached the edge of the Petri dish: *Fusarium graminearum* (**A**), *Fusarium solani* (**B**), *Fusarium oxysporum* (**C**). * represents a significant difference in volatiles from *C. iria* leaves between the control and the treatment at *P* ≤ 0.05 according to a Student’s *t*-test. Seven replicates of each treatment and three replicates of the control for each fungal species were conducted.

Based on the fungal-inhibition assay, volatiles emitted from the leaves of *C. iria* (at 70 days after germination) significantly inhibited the mycelial growth of *Fusarium graminearum*, with an inhibition rate of 8.96% (Figure 6A). Interestingly, volatiles emitted from *C. iria* did not inhibit growth of other fungal species in *Fusarium* (Figure 6B,C). Many terpenoid compounds identified to be constitutively emitted from *C. iria* leaves have been reported to have an anti-fungal effect on various species [9,10,11]. For example, ethanolic and hexane extracts from the aerial parts of *Juniperus lucayana* Britton, with sesquiterpenes as the dominant compounds, were documented to have an antifungal effect on *Botrytis cinerea* [28].

Pathogenic *Fusarium* species are widely distributed and can cause devastating losses in rice production. Compared to the susceptibility of rice to infection with *Fusarium* [29,30], *C. iria* appears to be more resistant [31]. In this study, we chose chopped fresh leaves instead of organic extracts to explore the antifungal effect of the volatiles emitted from *C. iria* leaves, which were shown to have an inhibitory effect on *F. graminearum*. This provides one possible explanation for the different pathogenicity of *Fusarium* to rice and *C. iria* plants, and points to a possible application of *C. iria* as a source of fungicide for the control of certain pathogenic *Fusarium* spp. and other fungal pathogens.

## 3. Materials and Methods

### 3.1. Plant Material

Seeds of *C. Iria* were kindly provided by Dr. Jacqueline Bede. Plants of *C. iria* were grown from seeds and kept in a climate-controlled incubator under the following conditions: temperature: day/night: 22 °C; photoperiod: 9/15; and illumination: 100 μE·m^−2^·s^−1^. Plants were produced in individual glass bottles with topsoil and kept continuously moist by maintaining the plants in 2 to 5 cm of water as previously reported [20].

### 3.2. Characterization of Terpenoids with Organic Extraction

At 20 days after germination, plant leaves were frozen in liquid nitrogen and ground into powder. Ethyl acetate was added (1 mL per 0.2 g tissue), and 1-octanol was added (0.003% w/v) as an internal standard. Extraction proceeded for 2 h at room temperature with continuous shaking. Five microliters of extract were injected into a GC-MS for separation and identification of the terpenoids compounds.

### 3.3. GC-MS Analysis

Terpenoids from organic extractions were analyzed on a Shimadzu 17A gas chromatograph coupled to a Shimadzu QP5050A quadrupole mass selective detector. Separation was performed on a Restek SHR5XLB column (30 m × 0.25 mm i.d. × 0.25 mm thickness) under the following conditions: helium was the carrier gas (flow rate of 5 mL min^−1^), a splitless injection (injection injector temperature = 250 °C) was used, and a temperature gradient of 5 °C /min from 40 °C (3 min hold) to 240 °C was applied. Terpenoid products were identified using the National Institute of Standards and Technology (NIST) mass spectral database and by comparison of retention times and mass spectra with authentic reference compounds purchased from Sigma-Aldrich (St. Louis, MO, USA). Quantification was performed as previously reported [26,32]. Representative single-ion peaks of each compound were integrated and compared with the equivalent response of the internal standard (single-ion method).

### 3.4. Temporal and Spatial Distribution of Terpenoids

Immature plants were assayed by organic extraction and GC-MS analysis (as described above) every 10 days during 20 to 100 days after germination. At approximately 110 days, the bracts opened, exposing the inflorescence. Plants at this stage were defined as mature and assayed every 10 days during the period of 110 to 150 days after germination. Immature plants were divided into aerial tissues (leaves) and subterranean tissues (roots). After flowering, mature plants were divided into root, leaf, and inflorescence (comprised of multiple spikes, flowers and nutlets) tissues. The concentration of monoterpenoids and sesquiterpenoids in the plant refers to the amount extracted per gram of plant tissue.

### 3.5. Chemical Treatments

Twenty-day-old plants were irrigated with 100 mL of water (control), 5 mM SA, Polyethylene Glycol 8000 (PEG-8000) at 20% w/v, or 5 mM MeJA, individually for 3 consecutive days at 10.00 am. All chemicals were purchased from Sigma-Aldrich (St. Louis, MO, USA). Organic extraction was performed 24 h after the last irrigation.

### 3.6. Insect Infestation and Physical Wounding

Beet armyworm (*Spodoptera exigua*) and FAW (*Spodoptera frugiperda*) were used as model herbivores to infest 20-day-old plants. Eggs and newly emerged larvae of FAW and BAW were transferred from glass jars (in which adults were contained to mate and lay eggs) to 37.5 mL cups containing approximately 15 to 20 mL of a pinto bean-based artificial diet as a food source. These diet cups containing larvae were maintained in an incubator (24 C, with 16 h light and 8 h dark). For plant treatments, BAW and FAW larvae were removed from the diet cups and placed on the leaves of plants at 4.00 pm. After 18 h (when approximately 20% of the leaf area had been consumed), insects were removed and the plants were used for headspace volatile collection. For physical wounding, leaves were cut with a sterile razor blade to produce two lateral incisions on each side of the midvein.

### 3.7. Headspace-Collection of Volatiles by Insect Infestation and Physical Wounding

Volatiles emitted from FAW-damaged, BAW-damaged, physically wounded, and control plants were collected in an open headspace sampling system (Analytical Research Systems, Gainesville, FL, USA) as previously described [21,22]. Volatiles were collected for 4 h by pumping air at a flow rate of 0.8 L/min through a SuperQ volatile collection trap. Volatiles were eluted with 100 μL of CH_2_Cl_2_, and 1-octanol was added as an internal standard for quantification. Five microliters of elute were injected into a GC-MS for separation and identification of the terpene compounds.

### 3.8. Anti-Fungal Effect of Volatiles Emitted from Leaves

Chopped fresh leaves (0.1 g) of 70-day-old *C. iria* were placed on the lid of Petri dishes, with potato dextrose agar (PDA) as culture medium on the dish bottom. The PDA plates had each been inoculated with one of three fungal species in *Fusarium*, including *Fusarium oxysporum*, *F. graminearum* and *F. solani* (collection of Dr. Bonnie Ownley, Department of Entomology and Plant Pathology, University of Tennessee). The inverted Petri dishes were incubated at 24 °C and the diameter of each fungal colony was recorded daily until the mycelium reached the edge of the Petri dish. There were seven replicates of each treatment and three replicates of the control for each fungal species.

### 3.9. Hierarchical Clustering and Data Visualization

The concentrations of individual sesquiterpenoids that accumulated in leaves from different treatments were used in hierarchical cluster analysis, which was conducted using Cluster 3.0 software (Version 1.39; Stanford University, Palo Alto, CA, USA) using average linkage analysis. Before the cluster analysis, procedures of “filter data” (with settings of % present ≥ 80, SD (Gene Vector) = 2, at least X observations with absolute value ≥ 2, maximum value − minimum value ≥ 2) and “adjust data” were carried out [26,27]. Heat maps were created using the Java TreeView 1.60 software (Stanford University, Stanford, CA, USA).

### 3.10. Statistical Analysis

Analyses of the concentration of terpenoid for each treatment and control were conducted using SAS (Version 8.02; SAS Institute, Cary, NC, USA) based on three biological replicates (volatiles collected from independent flower samples) and two technical replicates (repeat run of the same volatile sample on GC-MS); SEs were calculated for all mean values. Significant differences between treatments were calculated with a Tukey’s test at *P* ≤ 0.05.

## 4. Conclusions

In this study, we showed that the weed *C. iria* produces a diverse mixture of volatile terpenoids, including both monoterpenoids and sesquiterpenoids. Although production of terpenoids is developmentally regulated, accumulation of production of monoterpenoids and sesquiterpenoids exhibited temporal and spatial specificity. The production of terpenoids is also affected by environmental stresses, including both biotic and abiotic stresses. Treatment with methyl jasmonate and drought both significantly promoted accumulation of sesquiterpenoids in leaves. Infestation by beet armyworm (*Spodoptera exigua*) significantly promoted emission of total sesquiterpenoids, and induced production of more monoterpenoids and sesquiterpenoids specifically. Furthermore, volatiles from *C. iria* leaves had an anti-fungal effect on *Fusarium graminearum*. By analyzing the accumulation/emission patterns of volatile terpenoids, the terpene synthase genes responsible for their biosynthesis can be predicted, which may guide future efforts to characterize the molecular basis of terpenoid biosynthesis in *C. iria*.

## Figures and Tables

**Table 1:** The composition of terpenoids in *C. iria* leaves induced by biotic and abiotic stresses.

Compounds	Control L(O) ^a^	PEG L(O)	SA L(O)	MeJA L(O)	Control L(H) ^b^	FAW L(H)	BAW L(H)	PW L(H)
	Concentration (µg/g Fresh Weight)				Emission Rates (μg/h/g Fresh Weight)			
**Monoterpene**								
Limonene	ND ^c^	ND	ND	ND	ND	ND	0.15 ± 0.02	ND
β-Ocimene	ND	ND	ND	ND	ND	ND	0.31 ± 0.06	ND
Linalool	ND	ND	ND	0.94 ± 0.17	ND	ND	0.12 ± 0.01	ND
**Sesquiterpene**								
β-Elemene *	1.2 ^d^ ± 0.2a ^e^	2.0 ± 0.2a	1.5 ± 0.2a	6.8 ± 0.8b	0.051 ± 0.009a	0.21 ± 0.02a	1.1 ± 0.1b	0.11 ± 0.03a
α-Gurjunene	ND	ND	ND	ND	ND	ND	0.37 ± 0.05	ND
E-β-Caryophyllene *	6.7 ± 0.8a	13 ± 1b	8.9± 1.1a	39 ± 3c	0.45 ± 0.08a	1.2 ± 0.1b	6.7 ± 0.5c	0.88 ± 0.13b
α-Humulene *	1.7 ± 0.2a	3.6 ± 0.6b	2.4 ± 0.4a	10± 1c	0.084 ± 0.016a	0.33 ± 0.05a	1.7 ± 0.1b	0.13 ± 0.03a
α-Bergamotene	0.75 ± 0.12a	1.7 ± 0.2b	0.94 ± 0.08a	4.2 ± 0.7c	0.031 ± 0.007a	0.15 ± 0.02a	0.57 ± 0.04c	0.22 ± 0.04b
Germacrene D	ND	ND	ND	ND	ND	ND	0.038 ± 0.006	ND
α-Farnesene *	1.3 ± 0.2a	2.4 ± 0.4b	1.4 ± 0.3a	5.5 ± 0.4c	0.14 ± 0.02a	0.12 ± 0.02a	0.69 ± 0.09b	0.52 ± 0.06b
Elemol	0.59 ± 0.04a	0.91 ± 0.14b	0.56 ± 0.04a	2.4 ± 0.3c	0.032 ± 0.008a	0.042 ± 0.002a	0.075 ± 0.007a	0.091 ± 0.019a
Nerolidol	ND	ND	ND	2.73 ± 0.43	ND	ND	ND	ND
Hedycaryol	24 ± 4a	34 ± 3b	25 ± 4a	107 ± 11c	1.1 ± 0.1b	1.4 ± 0.2b	4.8 ± 0.6c	0.11 ± 0.02a
γ-Eudesmol	0.14 ± 0.02a	0.72 ± 0.08b	0.17 ± 0.04a	1.1 ± 0.2c	0.011 ± 0.002a	0.074 ± 0.010b	0.045 ± 0.004b	0.015 ± 0.003a
β-Eudesmol	0.25 ± 0.03a	1.2 ± 0.3a	0.34 ± 0.04a	2.4 ± 0.2b	0.013 ± 0.001a	0.13 ± 0.02c	0.045 ± 0.007b	0.030 ± 0.002b
α-Eudesmol	0.27 ± 0.04a	1.9 ± 0.3b	0.41 ± 0.05a	2.2 ± 0.2b	0.015 ± 0.004a	0.073 ± 0.010b	0.071 ± 0.006b	0.083 ± 0.013b
Total	37 ± 4a	61± 9b	41 ± 3a	185 ± 17c	1.9 ± 0.3a	3.6 ± 0.5a	16 ± 1b	2.5 ± 0.3a

^a^ O represents organic extraction. ^b^ H represents headspace volatile collection. ^c^ Not detected. ^d^ Means of three biological replicates and two technical replicates are presented. ^e^ Within each grouping for concentration or emissions rate, for each row, means ± SE followed by different letters represent a significant difference among treatments for a specific compound detected, according to Tukey’s test at *P* ≤ 0.05. * represents the terpenoid compounds that were identified by the authentic reference compounds.
